# Reclassification of Halomicroarcula saliterrae Straková et al. 2024 and Halomicroarcula onubensis Straková et al. 2024 into the genus Haloarcula, as Haloarcula saliterrae comb. nov. and Haloarcula onubensis comb. nov., respectively

**DOI:** 10.1099/ijsem.0.006510

**Published:** 2024-09-16

**Authors:** Dáša Straková, Cristina Sánchez-Porro, Rafael R. de la Haba, Antonio Ventosa

**Affiliations:** 1 Department of Microbiology and Parasitology, Faculty of Pharmacy, University of Sevilla, 41012 Sevilla, Spain

**Keywords:** *Haloarcula*, haloarchaea, *Halomicroarcula*, taxonomy, taxogenomic analysis

## Abstract

The haloarchaeal genera *Halomicroarcula* and *Haloarcula*, belonging to the family *Haloarculaceae*, order *Halobacteriales*, class *Halobacteria*, within the phylum *Methanobacteriota*, have previously exhibited significant phylogenetic and taxonomic overlaps. This issue was recently resolved by merging the two genera into a single genus, *Haloarcula*. However, *Halomicroarcula saliterrae* and *Halomicroarcula onubensis* were described almost simultaneously with the proposal to unify the genera *Haloarcula* and *Halomicroarcula*. Their names were validly published under the International Code of Nomenclature of Prokaryotes (ICNP) according to Validation List no. 217, alongside six *Haloarcula* species and the transfer of the existing *Halomicroarcula* species into the genus *Haloarcula*. Therefore a phylogenetic, phylogenomic, and comparative genomic analysis was carried out to clarify the taxonomic status of these two haloarchaeal species, *Halomicroarcula saliterrae* and *Halomicroarcula onubensis*, with lower priority than the six new species of the genus *Haloarcula*. Phylogenetic studies of 16S rRNA and *rpoB′* gene sequences, along with phylogenomic reconstructions using single-copy core-orthologous proteins, indicated that the two species clustered with the members of the genus *Haloarcula*. The overall genome relatedness indexes (OGRIs), comparative analyses of phenotypic features, and polar lipid profiles further supported their taxonomic reassignment as two separate species within the genus *Haloarcula*. Consequently, we propose the reclassification of *Halomicroarcula saliterrae* Straková *et al*. 2024 and *Halomicroarcula onubensis* Straková *et al*. 2024 into the genus *Haloarcula*, as *Haloarcula saliterrae* comb. nov. and *Haloarcula onubensis* comb. nov., respectively, in accordance with the ICNP.

## Introduction

The genus *Haloarcula* belongs to the family *Haloarculaceae*, within the order *Halobacteriales*, class *Halobacteria*, within the phylum *Methanobacteriota*. It was proposed by Torreblanca *et al*. in 1986 [1] by reassigning a species, previously described as *Halobacterium vallismortis*, to the new genus *Haloarcula*, as *Haloarcula vallismortis*. The genus *Halomicroarcula* was proposed in 2013 by Echigo *et al*. [2] to accommodate the species *Halomicroarcula pellucida*. The study by Durán-Viseras *et al*. in 2021 [3] suggested merging the genera *Halomicroarcula* and *Haloarcula* into a single genus, *Haloarcula*, based on phylogenomic similarities among their species. They recommended a more exhaustive study, encompassing a larger set of strains and additional genomic analyses, to better reflect the evolutionary relationships and phenotypic features of species of these genera. Since then, four novel *Halomicroarcula* species and eight new *Haloarcula* species have been published under the International Code of Nomenclature of Prokaryotes (ICNP). In January 2024, Ma *et al*. [4] conducted phylogenetic, phylogenomic, and comparative genomic analyses to reassess the taxonomic status of species of these two genera. Their key findings supported the merging of *Haloarcula* and *Halomicroarcula* into a single genus, *Haloarcula*, resulting in a more coherent classification within the family *Haloarculaceae*. Currently, the genus *Haloarcula* includes 27 species according to the List of Prokaryotic names with Standing in Nomenclature [5]: *Har. amylolytica* [6], *Har. amylovorans* [4], *Har. argentinensis* [7], *Har. halobia* [4], *Har. halophila* [4], *Har. hispanica* [8], *Har. japonica* [9], *Har. laminariae* [4], *Har. limicola* [4], *Har. litorea* [4], *Har. mannanilytica* [10], *Har. marina* [4], *Har. marismortui* [11], *Har. nitratireducens* [4], *Har. ordinaria* [4], *Har. pelagica* [4], *Har. pellucida* [4], *Har. rara* [4], *Har. rubra* [4], *Har. salina* [12], *Har. salinisoli* [4], *Har. sebkhae* [13], *Har. terrestris* [14], *Har*. *vallismortis* [1], *Har. quadrata* [15], *Har. salaria* [16], and *Har. tradensis* [16]. The last three species have recently been proposed as later heterotypic synonyms of *Har. argentinensis* and *Har. marismortui*, respectively [12,14].

On the other hand, two new *Halomicroarcula* species, *Halomicroarcula saliterrae* and *Halomicroarcula onubensis*, were described in 2024 by Straková *et al*. [17]. These species names were validly published under the ICNP according to Validation List no. 217 [18] concurrently with the six *Haloarcula* species described by Ma *et al.* [4] and the transfer of the existing *Halomicroarcula* species into the genus *Haloarcul*a. Therefore, a comprehensive phylogenetic, phylogenomic, and comparative genomic analysis was conducted to elucidate the taxonomic status of these two haloarchaeal species. Together with comparative analysis of phenotypic features and polar lipid profiles, this analysis confirmed that these species should be reclassified into the genus *Haloarcula*, as *Haloarcula saliterrae* comb. nov. and *Haloarcula onubensis* comb. nov.

## Methods

### Phylogenetic studies

The 16S rRNA and *rpoB′* gene sequences of the studied species, obtained from their respective genomes, were retrieved from the NCBI GenBank database and taxonomically associated with their phylogenetic neighbours using a blastn search [19]. Multiple sequence alignments of the 16S rRNA and *rpoB′* genes from *Halomicroarcula saliterrae* S1CR25-12^T^, *Halomicroarcula onubensis* S3CR25-11^T^, and their closest relatives were performed using the Fast Aligner tool in arb software [20]. The alignments were then visually inspected and corrected. Phylogenetic tree reconstructions based on the 16S rRNA and *rpoB′* gene sequences were conducted using the maximum-likelihood [21], neighbour-joining [22], and maximum-parsimony algorithms [23] implemented in arb software [20]. The ‘gitana’ script was used for formatting and visualizing the phylogenetic tree [24].

### Phylogenomic analyses and genomic indexes

For the taxogenomic analyses, publicly available genome sequences of *Halomicroarcula saliterrae* S1CR25-12^T^, *Halomicroarcula onubensis* S3CR25-11^T^, and closely related species were obtained from the NCBI GenBank database, adhering to the minimal standards for the use of genome data for taxonomic purposes [25,26]. The features of these genomes have been published previously [3,4, 17]. The Enveomics toolbox [27] was used to identify the clusters of orthologous proteins shared by all analysed species. The single-copy core-orthologous proteins were individually aligned using muscle version 5.1 [28]. To determine the phylogenomic position of *Halomicroarcula saliterrae* and *Halomicroarcula onubensis* relative to the species of the genus *Haloarcula* and other related haloarchaea, an approximately maximum-likelihood phylogenomic tree was reconstructed using FastTreeMP version 2.1.8 [29].

Overall genome relatedness indexes (OGRIs) were calculated among *Halomicroarcula saliterrae* S1CR25-12^T^, *Halomicroarcula onubensis* S3CR25-11^T^, and the species of the genus *Haloarcula* and other related species of the family *Haloarculaceae*. Orthologous average nucleotide identity (OrthoANI) values were determined by OrthoANIu tool version 1.2 [30], digital DNA–DNA hybridization (dDDH) values were obtained using the Genome-to-Genome Distance Calculator (GGDC 3.0) from the Leibniz Institute DSMZ (Germany) [31,32], and average amino acid identity (AAI) values were calculated using the ‘aai.rb’ script from the Enveomics collection [27].

### Comparative analysis of phenotypic features and chemotaxonomic profiles

A comparative analysis of phenotypic features, including morphological, physiological, biochemical, and nutritional characteristics, as previously reported [3,4, 12, 17], was conducted for *Halomicroarcula saliterrae*, *Halomicroarcula onubensis* and their closest related species.

In addition, the characterization and differentiation of haloarchaea at the genus level have significantly benefited from the analysis of polar lipids, which serve as valuable taxonomic markers [1,26, 33]. A comparative analysis of the lipid profiles of *Halomicroarcula saliterrae* and *Halomicroarcula onubensis*, as previously presented [17], with those of species of the genus *Haloarcula* was conducted to confirm the taxonomic affiliation of these two species within the genus *Haloarcula*.

## Results and discussion

### Phylogenetic studies incorporating six additional *Haloarcula* species

**Fig. 1.**
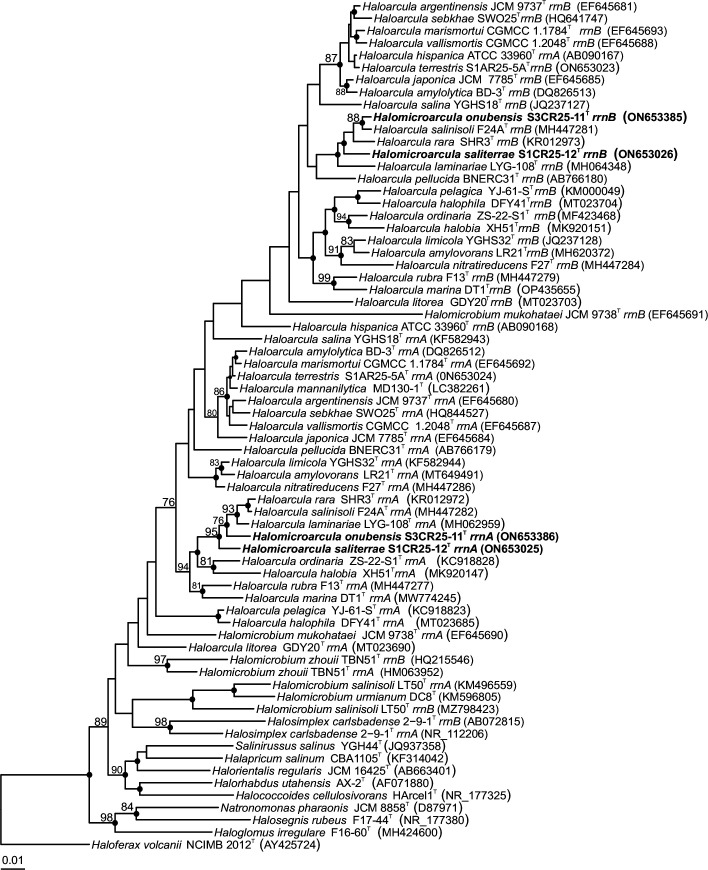
Neighbour-joining phylogenetic tree based on the 16S rRNA gene sequence comparison of the species *Halomicroarcula saliterrae* S1CR25-12^T^ and *Halomicroarcula onubensis* S3CR25-11^T^ with species of *Haloarcula* and other related species within the family *Haloarculaceae*. The species *Haloferax volcanii* NCIMB 2012^T^ was used as an outgroup. Sequence accession numbers are shown in parentheses. Bootstrap values ≥70% (based on 1000 pseudo-replicates) are shown at branch points. Filled circles indicate branches that were also recovered in the trees generated with the maximum-likelihood and maximum-parsimony algorithms. Bar, 0.01 expected substitutions per nucleotide position.

We present an extended phylogenetic analysis including all current species of *Haloarcula*, that incorporate the six recently described *Haloarcula* species not included in our previous study conducted on *Halomicroarcula saliterrae* and *Halomicroarcula onubensis* [17]. The additional six *Haloarcula* species described by Ma *et al.* [4] exhibited 16S rRNA gene sequence identities ranging from 98.3 to 90.7%, and from 98.2 to 91.0% with *Halomicroarcula saliterrae* S1CR25-12^T^ and *Halomicroarcula onubensis* S3CR25-11^T^, respectively. Among the six additional *Haloarcula* species, *Haloarcula rara* SHR3^T^ was identified as the most closely related to both *Halomicroarcula* species. Additionaly, an analysis of the *rpoB′* gene sequences of *Halomicroarcula saliterrae* S1CR25-12^T^ and *Halomicroarcula onubensis* S3CR25-11^T^, compared with the *rpoB′* gene sequences of the six additional *Haloarcula* species, demonstrated that the closest relatedness was to *Haloarcula rara* SHR3^T^, with sequence identities of 93.6 and 92.5%, respectively. Phylogenetic reconstruction based on the 16S rRNA (Fig. 1) and *rpoB′* (Fig. S1, available in the online version of this article) gene sequences indicated the affiliation of *Halomicroarcula saliterrae* S1CR25-12^T^ and *Halomicroarcula onubensis* S3CR25-11^T^ within the genus *Haloarcula*.

### Phylogenomic analyses and genomic indexes validate reclassification to the genus *Haloarcula*

**Fig. 2.**
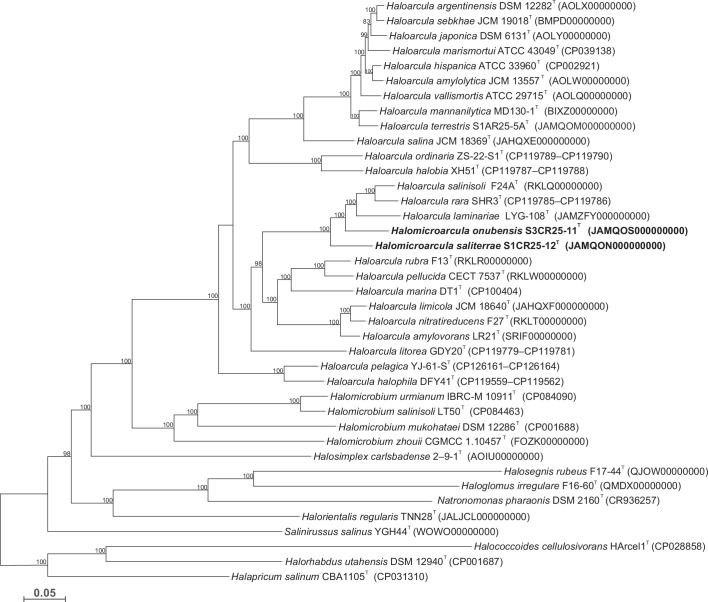
Approximately maximum-likelihood phylogenomic tree based on the comparison of 153 single-copy core-orthologous proteins showing the relationships of the species *Halomicroarcula saliterrae* S1CR25-12^T^ and *Halomicroarcula onubensis* S3CR25-11^T^ with species of *Haloarcula* and other related species within the family *Haloarculaceae*. Sequence accession numbers are shown in parentheses. Branch support values (%) are computed with the Shimodaira–Hasegawa test and are shown at branch points. Bar, 0.05 substitutions per amino acid position.

Similar to the 16S rRNA gene sequence analysis, the phylogenomic and comparative analyses of genomic identities were expanded to include the six recently described *Haloarcula* species. The approximately maximum-likelihood phylogenomic tree reconstruction, based on the comparison of 153 single-copy core-orthologous proteins, revealed that *Halomicroarcula saliterrae* S1CR25-12^T^ and *Halomicroarcula onubensis* S3CR25-11^T^ formed a cluster with *Haloarcula salinisoli* F24A^T^, *Haloarcula laminariae* LYG-108^T^, and *Haloarcula rara* SHR3^T^ (Fig. 2).

**Fig. 3.**
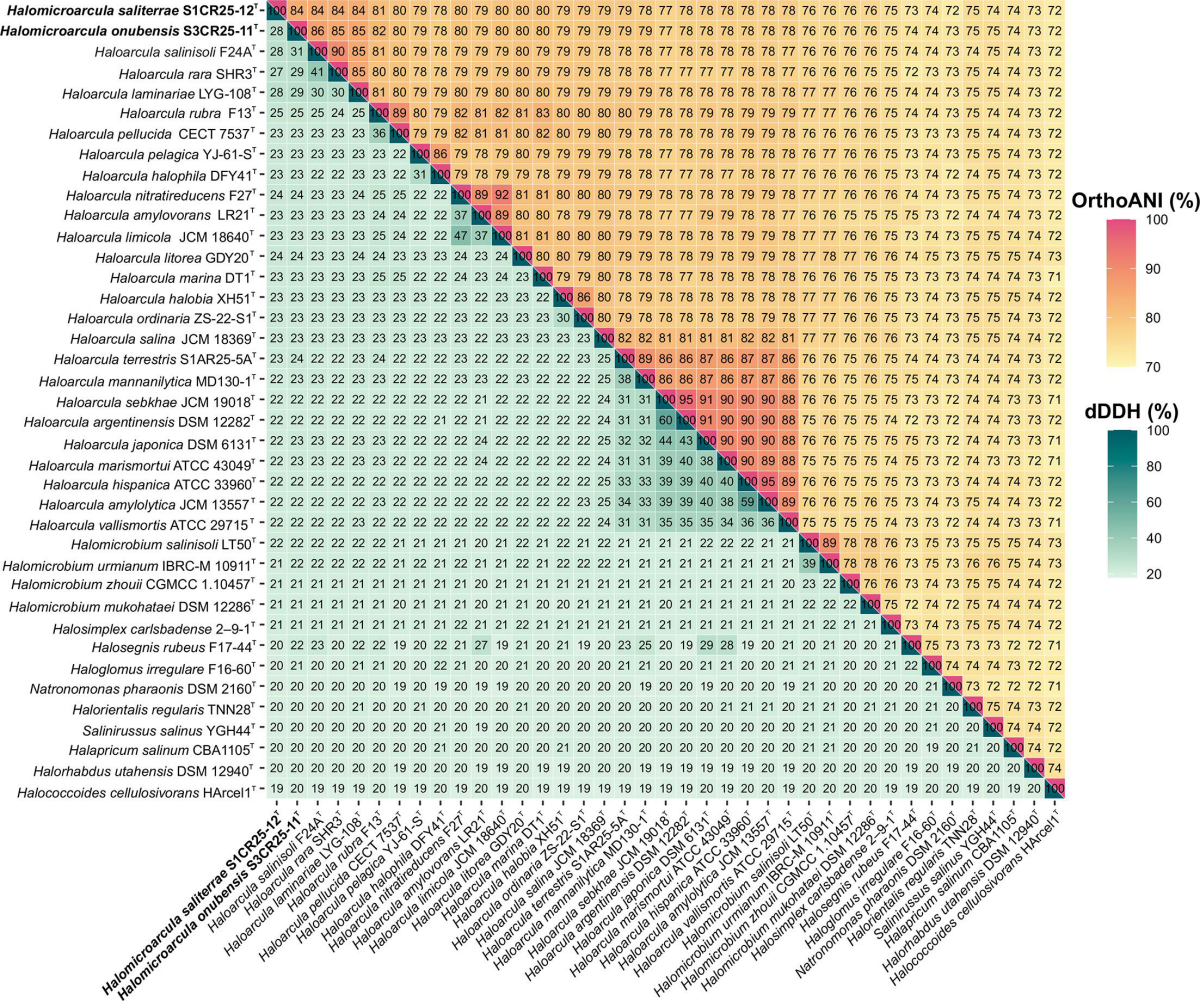
Heatmap displaying OrthoANI (upper right) and dDDH (lower left) percentages among the species *Halomicroarcula saliterrae* S1CR25-12^T^ and *Halomicroarcula onubensis* S3CR25-11^T^, and members of the genus *Haloarcula* and other related species of the family *Haloarculaceae*.

Additionally, to clarify the taxonomic positions of *Halomicroarcula saliterrae* S1CR25-12^T^ and *Halomicroarcula onubensis* S3CR25-11^T^, we calculated the OGRIs among the species of the family *Haloarculaceae*. The OrthoANI, dDDH, and AAI values among these two species and the current species of the genus *Haloarcula* were 86.0–77.4%, 30.9–21.6%, and 84.2–70.1 %, respectively, all significantly below the threshold values for prokaryotic species delineation (OrthoANI 95–96%, dDDH 70%, AAI 95-96%) [34,37], and mostly above genus cutoff (AAI 65-72%)[38] (Figs3  4). These results indicate unequivocally that *Halomicroarcula saliterrae* S1CR25-12^T^ and *Halomicroarcula onubensis* S3CR25-11^T^ should be reclassified within the genus *Haloarcula*. These results support that the two species, *Halomicroarcula saliterrae* and *Halomicroarcula onubensis*, show genotypic features compatible with those of the species of *Haloarcula*.

**Fig. 4.**
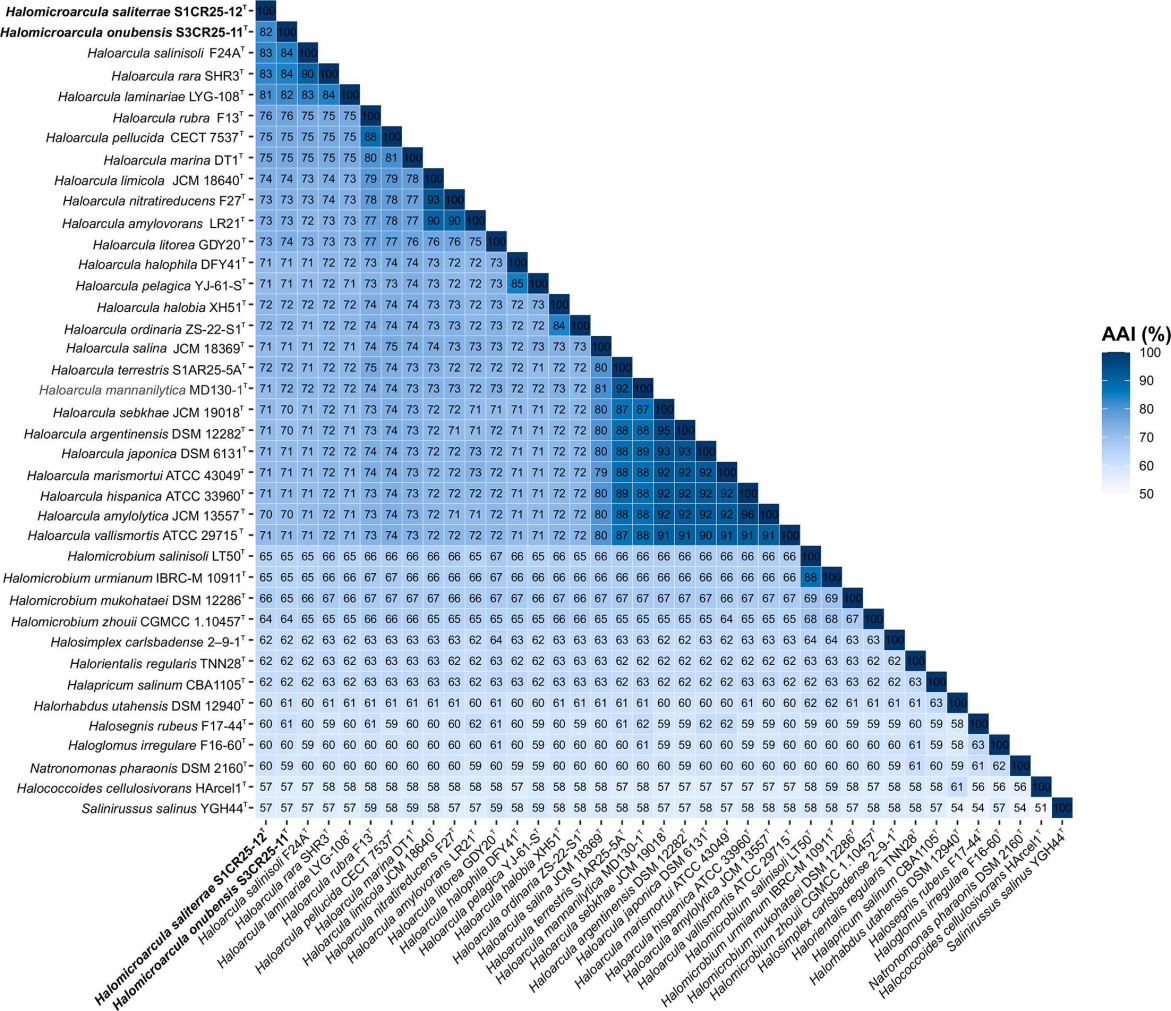
Heatmap showing AAI percentages among *Haloarcula* species, including the species *Halomicroarcula saliterrae* S1CR25-12^T^ and *Halomicroarcula onubensis* S3CR25-11^T^, and other related species of the family *Haloarculaeae*.

### Phenotypic features and chemotaxonomic analysis confirm the affiliation to the genus *Haloarcula*

A comparative analysis of the phenotypic features of *Halomicroarcula saliterrae* S1CR25-12^T^, *Halomicroarcula onubensis* S3CR25-11^T^, and their phylogenetically closest species, *Haloarcula laminariae* LYG-108^T^, *Haloarcula salinisoli* F24A^T^, and *Haloarcula rara* SHR3^T^, as well as the type species of the genus, *Haloarcula vallismortis* ATCC 29715^T^, was conducted based on previous data [3,4, 12, 17, 39]. Their phenotypic characteristics are fully compatible with those of species of the genus *Haloarcula*. However, they presented some different characteristics, as shown in Table 1.

**Table 1.** Differential characteristics between the species *Halomicroarcula saliterrae* S1CR25-12^T^ and *Halomicroarcula onubensis* S3CR25-11^T^, and related species of the genus *Haloarcula*

Characteristic	*Hma*. *saliterrae* S1CR25-12^T^*	*Hma*. *onubensis* S3CR25-11^T^*	*Har. rara* SHR3^T†^	*Har. laminariae* LYG-108^T‡^	*Har. salinisoli* F24A^T§^	*Har. vallismortis* ATCC 29715^T¶^
Morphology	Pleomorphic rods	Pleomorphic rods	Pleomorphic	Rods	Rods	Pleomorphic
Colony pigmentation	Red	Orange red	Red	Red	Pink	Pink
NaCl requirement:						
Range (%, w/v)	15–30	12–30	8–28	8–28	15–30	>15
Optimum (%, w/v)	25	25	18	15	25	25
Temperature requirement:						
Range (°C)	20–50	25–55	25–55	20–50	25–50	20–45
Optimum (°C)	37	37	37	40	37	40
pH requirement:						
Range	6.0–9.0	6.0–9.0	5.5–8.5	5.0–9.5	6.0–8.5	5.5–8.5
Optimum	7.0–8.0	7.0–8.0	7.0	7.0	7.0–7.5	7.4–7.5
Hydrolysis of:						
Gelatin	−	−	−	−	+	−
Tween 80	−	−	+	−	+	−
Reduction of nitrite	−	−	−	−	+	−
Utilization as sole carbon and energy source:						
Citrate	−	−	+	−	+	nd
d-Galactose	+	−	+	+	−	+
d-Sorbitol	−	−	−	+	+	nd
Fumarate	−	+	+	+	+	nd
Glycerol	−	−	+	+	−	+
Sucrose	−	+	+	+	+	+
Lactose	−	−	+	−	−	−
d-Xylose	−	+	−	−	−	−
Maltose	+	−	−	−	+	+
Glycine	+	−	−	+	nd	nd
l-Alanine	+	−	−	−	nd	nd

*Data from Straková *et al.* [17].

†Data from Ma *et al.* [4].

‡Data from Ma *et al.* [12].

§Data from Durán-Viseras *et al.* [3].

¶Data from Gonzalez *et al.* [39].

nd, Not determined.

Furthermore, a comparative chemotaxonomic analysis of *Halomicroarcula onubensis* and *Halomicroarcula saliterrae* was conducted to confirm their affiliation within the genus *Haloarcula*. The major polar lipids of the two species included phosphatidylglycerol, phosphatidylglycerol phosphate methyl ester, phosphatidylglycerol sulfate, and a glycolipid chromatographically identical to sulfated diglycosyl diether [17]. These polar lipid profiles correspond to the currently established lipid patterns observed in species of the genus *Haloarcula* [4], validating the taxonomic reclassification of *Halomicroarcula saliterrae* and *Halomicroarcula onubensis* within this genus.

## Taxonomic conclusions

The species names *Halomicroarcula saliterrae* and *Halomicroarcula onubensis* were validly published under the ICNP according to Validation List no. 217 [18], concurrently with the six *Haloarcula* species described by Ma *et al.* [4] that also proposed the unification of the genera *Haloarcula* and *Halomicroarcula*. Section 7 of the ICNP [40] addresses changes in names of taxa as a result of transference, union, or change in rank, with actions depending on taxonomic judgement. With this study we confirm and support the proposal by Ma *et al.* [4] about the unification of the genera *Haloarcula* and *Halomicroarcula*. Moreover, following the phylogenetic, phylogenomic, and comparative genomic analysis to ascertain their taxonomic status, together with a comparative examination of phenotypic features and polar lipid profiles, we confirmed that *Halomicroarcula saliterrae* and *Halomicroarcula onubensis* should not be considered as separate species of another genus, since they belong to the expanded genus *Haloarcula*. Consequently, we propose the reclassification of *Halomicroarcula saliterrae* Straková *et al*. 2024 and *Halomicroarcula onubensis* Straková *et al*. 2024 into the genus *Haloarcula*, as *Haloarcula saliterrae* comb. nov. and *Haloarcula onubensis* comb. nov., respectively.

## DESCRIPTION OF *HALOARCULA SALITERRAE* COMB. NOV.

*Haloarcula saliterrae* (sa.li.ter’rae. L. masc. n. *sal*, salt; L. fem. n. *terra*, land; N.L. gen. n. *saliterrae*, of saline soil).

Basonym: *Halomicroarcula saliterrae* Straková *et al*. 2024.

The description is identical to that given for *Halomicroarcula saliterrae* by Straková *et al*. 2024.

The type strain is S1CR25-12^T^ (=CECT 30620^T^=CCM 9252^T^).

## DESCRIPTION OF *HALOARCULA ONUBENSIS* COMB. NOV.

*Haloarcula onubensis* (o.nu.ben’sis. L. fem. adj. *onubensis*, of or belonging to Onuba, the ancient Latin name of Huelva, a city in Spain, where the type strain was isolated).

Basonym: *Halomicroarcula onubensis* Straková *et al*. 2024.

The description is identical to that given for *Halomicroarcula onubensis* by Straková *et al*. 2024.

The type strain is S3CR25-11^T^ (=CECT 30621^T^=CCM 9254^T^).

## Supplementary material

10.1099/ijsem.0.006510 Uncited Fig. S1.

## Abbreviations

AAI: average amino acid identity
dDDH: digital DNA–DNA hybridization
ICNP: International Code of Nomenclature of Prokaryotes
OGRI: overall genome relatedness index
OrthoANI: orthologous average nucleotide identity
