# Supplementary material for: Reclassification of Halomicroarcula saliterrae Straková et al. 2024 and Halomicroarcula onubensis Straková et al. 2024 into the genus Haloarcula, as Haloarcula saliterrae comb. nov. and Haloarcula onubensis comb. nov., respectively
Source: Int J Syst Evol Microbiol. 2024 Sep 16;74(9):006510. doi: 10.1099/ijsem.0.006510 (PMC12453561; doi:10.1099/ijsem.0.006510)
Supplement: Uncited Fig. S1. [file ijsem-74-06510-s001.pdf]

Supplementary Material

Reclassification of *Halomicroarcula saliterrae* Straková et al. 2024 and *Halomicroarcula onubensis* Straková et al. 2024 to the genus *Haloarcula*, as *Haloarcula saliterrae* comb. nov. and *Haloarcula onubensis* comb. nov., respectively

Dáša Straková, Cristina Sánchez-Porro, Rafael R. de la Haba and Antonio Ventosa

Department of Microbiology and Parasitology, Faculty of Pharmacy, University of Sevilla, 41012 Sevilla, Spain

***Correspondence:** Antonio Ventosa, ventosa@us.es

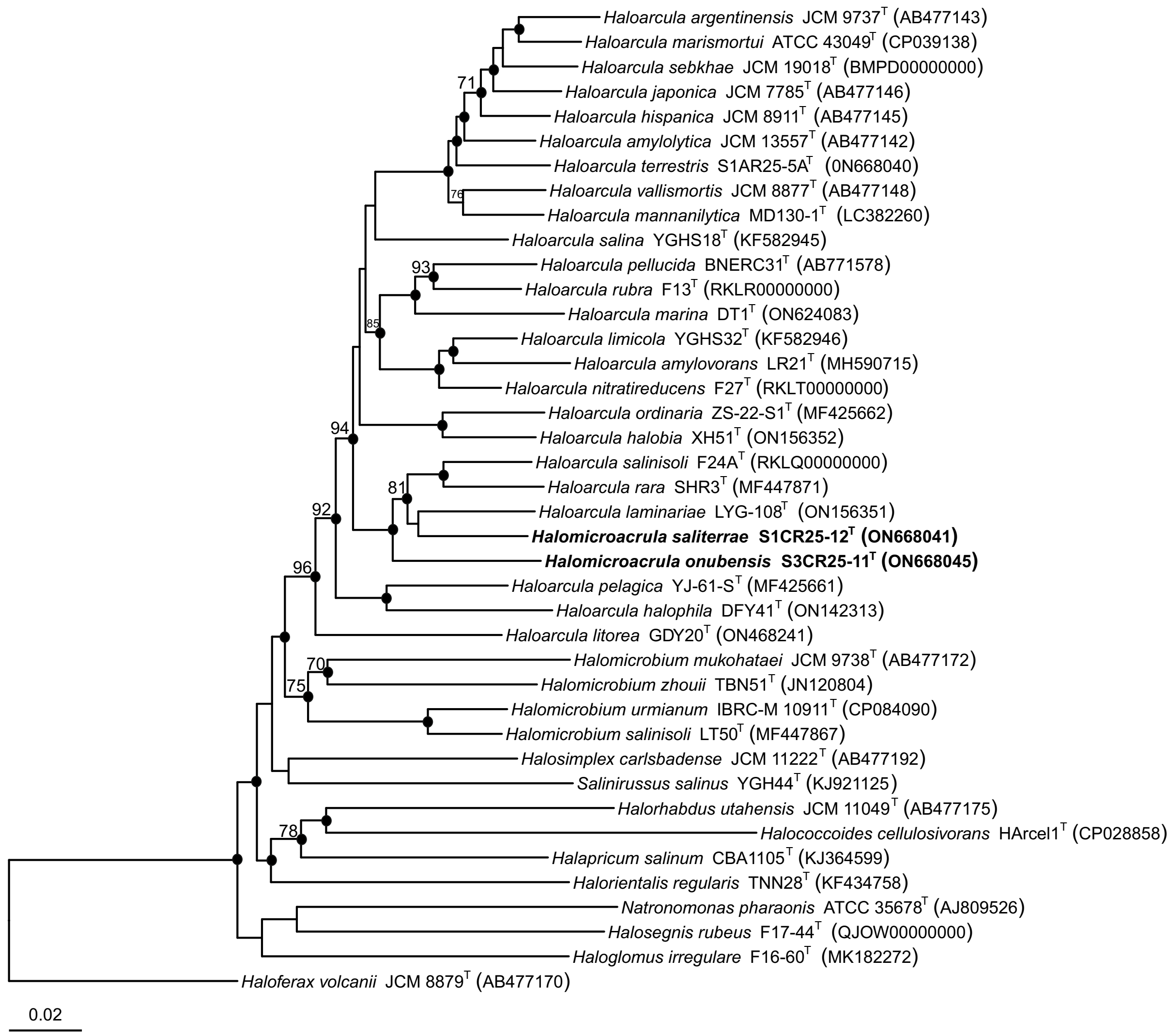

Supplementary Figure S1. Neighbor-joining phylogenetic reconstruction based on *rpoB'* gene sequences of the species *Halomicroarcula saliterrae* S1CR25-12^{T} and *Halomicroarcula onubensis* S3CR25-11^{T}, and related species of the family *Haloarculaceae*. The species *Haloferax volcanii* JCM 8879^{T} was used as an outgroup. Sequence accession numbers are shown in parentheses. Bootstrap values (%) higher than 70% are indicated at branch points. Filled circles indicate that the corresponding nodes were obtained in the trees generated with the neighbor-joining and maximum-likelihood algorithms. Bar, 0.02 expected substitutions per nucleotide position.
